# Serum Dioxin, Testosterone, and Subsequent Risk of Benign Prostatic Hyperplasia: A Prospective Cohort Study of Air Force Veterans

**DOI:** 10.1289/ehp.8957

**Published:** 2006-07-20

**Authors:** Amit Gupta, Norma Ketchum, Claus G. Roehrborn, Arnold Schecter, Corinne C. Aragaki, Joel E. Michalek

**Affiliations:** 1 Department of Urology, The University of Texas Southwestern Medical Center at Dallas, Dallas, Texas, USA; 2 The University of Texas School of Public Health, Regional Campus at Dallas, Dallas, Texas, USA; 3 Air Force Research Laboratory, Brooks City-Base, Texas, USA; 4 Center for Epidemiology and Biostatistics, University of Texas Health Science Center at San Antonio, San Antonio, Texas, USA

**Keywords:** benign prostatic hyperplasia, BPH, dioxins, endocrine disruptors, persistent organic pollutants, Ranch Hand, testosterone, TCDD, Vietnam veterans

## Abstract

**Background:**

Operation Ranch Hand veterans were involved in spraying herbicides, including Agent Orange, during the Vietnam War in 1962–1971; Agent Orange was contaminated with 2,3,7,8-tetrachlorodibenzo-*p*-dioxin (TCDD). It has been hypothesized that dioxins may be partially responsible for an increase of male reproductive tract disorders such as testicular cancer, cryptorchidism, and hypospadias.

**Objectives:**

In this study, our objective was to assess the effect of serum TCDD concentration on the risk of development of benign prostatic hyperplasia (BPH) and on serum testosterone levels.

**Methods:**

This study was a longitudinal, prospective cohort study made up of U.S. Air Force veterans involved in Operation Ranch Hand. Other Air Force veterans who did not spray herbicides were included as comparisons. BPH was determined by medical record review and by medical examinations conducted during the study. Data were available for 971 Ranch Hand and 1,266 comparison veterans. We investigated the relationship between BPH and serum TCDD level using the Cox proportional hazards models adjusted for testosterone levels, body mass index (BMI), and the percentage change in BMI per year.

**Results:**

In univariate and multivariate analyses, the risk of BPH decreased with increasing serum TCDD in the comparison group. The multivariate risk ratio for BPH in the comparison group was 0.84 (95% confidence interval, 0.73–0.98). Excluding men with prostate cancer, inflammatory or other prostatic diseases did not substantially alter the association. Serum testosterone levels were inversely associated with serum TCDD levels in both Ranch Hand and comparison groups.

**Conclusions:**

TCDD exposure at general population levels is associated with a decreasing risk of BPH with higher exposure levels. TCDD exposure is also negatively associated with serum testosterone levels.

Dioxins [polychlorinated dibenzo-*p*-dioxins (PCDDs)] belong to a group of chemicals identified as persistent organic pollutants (POPs) because of their tendency to resist degradation and persist in the environment. There are 75 possible PCDD congeners, of which 7 are most often found in the human body. 2,3,7,8-Tetrachlorodibenzo-*p*-dioxin (TCDD) is the prototype dioxin congener and is the most toxic dioxin. Dioxins are “endocrine-disrupting” chemicals. They enter the body through ingestion of contaminated food; dioxins accumulate in body lipids of living organisms and magnify as they move up the food chain. Dioxins have a long half-life; the half-life of TCDD is estimated to be between 7 and 11 years.

The incidence of disorders of the male reproductive tract such as testicular cancer, cryptorchidism, and hypospadias has increased over the past decades. It has been hypothesized that dioxins may be partially responsible for this increase (Toppari et al. 1996). However, evidence supporting this hypothesis has come largely from animal studies; a few human studies have provided limited evidence. Hardell et al. (2003) reported that levels of certain polychlorinated biphenyls (PCBs) were higher in mothers of men with testicular cancer compared with controls. Some PCBs are dioxin-like and produce responses similar to dioxins. Egeland et al. (1994) found an inverse relation between serum TCDD levels and serum testosterone in chemical production workers. Men prenatally exposed to PCBs and polychlorinated dibenzofurans (PCDF) in the Yucheng rice oil poisoning had a higher percentage of sperm with abnormal morphology, lower sperm motility, and decreased capacity of sperm to penetrate hamster oocytes (Guo et al. 2000). Transplacentally exposed children also had shorter penises at 11–14 years of age (Guo et al. 1993). We have also reported an inverse association between serum dioxin levels and benign prostatic hyperplasia (BPH) in a cross-sectional study (Gupta et al. 2006). We found that men with higher dioxin levels had lower odds of having BPH. In the present study, a longitudinal study of Vietnam War veterans, we tested the hypothesis that higher TCDD exposure leads to a lower risk of being diagnosed with BPH. We also studied the association between TCDD exposure and serum testosterone.

## Materials and Methods

The Air Force Health Study is a prospective study of Operation Ranch Hand veterans and a comparison group of other Air Force veterans designed to assess the effects of exposure to Agent Orange and its TCDD contaminant during the Vietnam War. Operation Ranch Hand veterans were involved in spraying herbicides (including Agent Orange), and TCDD was a contaminant in Agent Orange. The comparison group was composed of other Air Force veterans involved in aircraft missions not involving herbicide spraying in Southeast Asia in the same period (1962–1971) during which the Ranch Hand group was active. The comparison and Ranch Hand groups were matched on age, race, and occupation in the military. The study involved comprehensive medical examinations conducted in 1982, 1985, 1987, 1992, 1997, and 2002, along with regular review and coding of medical records. Details of the study design and methodology have been previously published (Wolfe et al. 1990).

Serum TCDD levels were measured for most veterans in 1987 at the Centers for Disease Control and Prevention (Atlanta, GA) using high-resolution gas chromatography and high-resolution mass spectrometry; TCDD levels were reported in parts per trillion on a lipid weight basis (Patterson et al. 1987). For those veterans for whom TCDD levels were measured after 1987 (*n* = 295), the TCDD levels were extrapolated to 1987 using a first-order kinetics model with a constant half-life of 8.7 years (Michalek et al. 1996). Nondetectable TCDD levels were replaced by the limit of detection divided by √2. The TCDD exposure in the comparison group is equivalent to the background exposure in the general population. According to the National Human Adipose Tissue Survey (NHATS), the mean TCDD body burden for U.S. men was 4.22 ppt compared with a mean TCDD level of 4.6 ppt in the comparison group (Orban et al. 1994).

Prostatic conditions such as BPH, prostate cancer, inflammatory prostatic diseases, and other prostatic diseases were coded from medical records according to the *International Classification of Diseases and Related Problems*, *Ninth Revision* [World Health Organization (WHO) 1979]. The occurrence of BPH and prostate cancer were determined by medical record review, which included the records from the veteran’s personal physician and the medical examinations conducted as part of the study. The date of onset was defined as the date of first diagnosis. Serum total testosterone was measured by radioimmunoassay in serum collected in the morning after an overnight fast. Medication use was elicited by interview and verified by medical record review.

We calculated the percentage change in body mass index (BMI) per year as [(BMI_1987_ − BMI_tour_) ÷ (BMI_tour_ × years since end of tour until 1987)] × 100, where BMI_1987_ is the BMI measured in the 1987 examination cycle and BMI_tour_ is the BMI at the end of the Southeast Asia tour.

Participants were included in the analysis if the serum TCDD level was measured (207 comparison veterans and 99 Ranch Hand veterans were excluded); testosterone level was measured in 1987 (166 comparison veterans and 44 Ranch Hand veterans were excluded); BMI at the end of the Southeast Asia tour was available (3 comparison veterans were excluded); BPH outcome information was available (7 comparison veterans and 7 Ranch Hand veterans were excluded); and if participants were not taking testosterone medications (1 comparison veteran and 1 Ranch Hand veteran were excluded). The analytical cohort comprised 1,266 comparison veterans and 971 Ranch Hand veterans who were followed up to 6 August 2004, after which the data were censored.

### Statistical analysis

Serum TCDD and testosterone levels were log-transformed because they were not normally distributed. Serum TCDD levels were also divided into quartiles. We used the Cox proportional hazards regression model to calculate the multivariate relative risk (RR) for the diagnosis of BPH. Time to BPH diagnosis was the dependent variable and was calculated as the time from birth to the date of BPH diagnosis, death, or 6 August 2004, whichever was earlier. Covariates were serum TCDD and testosterone levels [natural log (ln)-transformed], BMI at the 1987 examination, and the percentage change in BMI per year.

We used multivariate linear regression analysis to model the relationship between serum testosterone and TCDD levels. ln-Transformed serum testosterone level in 1987 was the dependent variable. The predictor variables were serum TCDD level (ln-transformed), age, BMI at the time of testosterone measurement, and the percentage change in BMI per year.

We conducted a sensitivity analysis to outcome definition. The association between serum TCDD levels and the risk of BPH was studied by excluding the following from the study population: *a*) men with history of prostate cancer; *b*) men with a history of prostate cancer, inflammatory prostatic diseases, or other prostatic diseases; *c*) men who developed BPH before 1988 (serum TCDD was measured for most veterans in 1987, and this would allow assessment of exposure before assessment of the disease status); *d*) men who developed BPH before 1991 (to allow a minimum of 3 years between exposure and outcome assessment); *e*) men who developed BPH before 1994 (to allow a minimum of 6 years between exposure and outcome assessment); and *f*) men who had a history of prostate cancer, inflammatory prostatic diseases, or other prostatic diseases or who developed BPH before 1994.

All significance tests were two sided with a significance level of α ≤ 0.05. All statistical analyses were performed using SAS, version 8.02 (SAS Institute Inc., Cary, NC).

## Results

Serum TCDD levels were higher in the Ranch Hand group (mean ± SD, 26.9 ± 45.5 ppt; median, 11.7 ppt; range, 0.6–617.8 ppt) than in the comparison group (mean ± SD, 4.6 ± 2.9 ppt; median, 4.1 ppt; range: 0.4–54.8 ppt). Descriptive characteristics of the comparison and Ranch Hand groups are presented in Table 1. The two groups were similar with respect to age, racial composition, serum testosterone levels, BMI, and the percentage change in BMI per year. The comparison and Ranch Hand veterans were divided into four quartiles based on serum TCDD levels (Table 2). At the time of censoring, 56% (705/1,259) of the comparison veterans and 57% (551/964) of the Ranch Hand veterans had been diagnosed with BPH.

We evaluated the risk of BPH in relation to the serum TCDD levels using the ln-transformation of TCDD levels as the predictor variable (Table 3). In univariate and multivariate analysis, the risk of BPH decreased with increasing serum TCDD levels in the comparison group, but appeared to increase in the Ranch Hand group. The multivariate RR for BPH in the comparison group was 0.84 [95% confidence interval (CI), 0.73–0.98]. This implies that the risk of being diagnosed with BPH decreases by 16% with exponential (2.72-fold) increase in serum TCDD levels.

**Figure 1:**
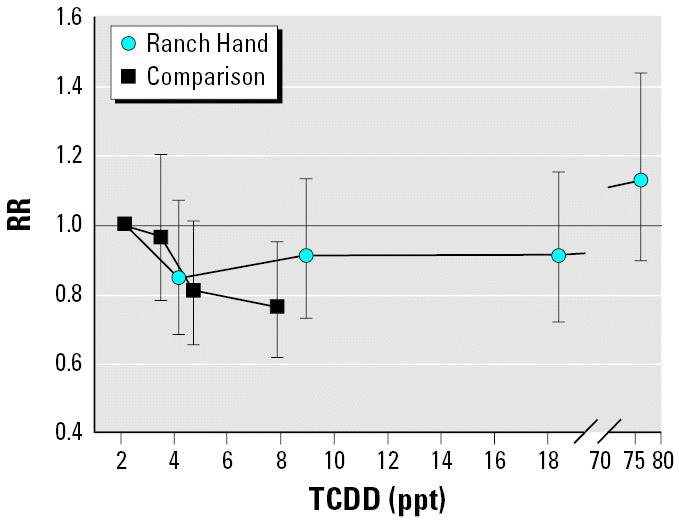
RR (95% CI) of developing BPH with increasing serum TCDD levels among comparison and Ranch Hand veterans in the Air Force Health Study. TCDD quartile I of the comparison veterans is the referent group for the Ranch Hand veterans.

We performed further analyses using serum TCDD quartiles (Table 3). The first quartile of the comparison veterans was used as the referent category for both the comparison and the Ranch Hand veterans. This was done in order to compare the results of the Ranch Hand veterans with those of the comparison veterans. In the comparison veterans the risk of being diagnosed with BPH decreased almost linearly with increasing TCDD exposure quartiles (test for trend = 0.049) (Table 3, Figure 1). In the Ranch Hand veterans, the highest TCDD quartile showed an increased risk of developing BPH compared with the first quartile of the comparison veterans, which was not statistically significant (Table 3). The data were also analyzed using the first quartile of the Ranch Hand veterans as the referent category for the other three quartiles of the Ranch Hand veterans. The relative risk of BPH was 1.07 (95% CI, 0.85–1.36; *p* = 0.55), 1.08 (95% CI, 0.85–1.39; *p* = 0.53), and 1.35 (95% CI, 1.05–1.74; *p* = 0.02) for the second, third, and fourth quartiles, respectively. We noted a trend (*p* = 0.11) toward increased risk of BPH that was confined completely to the fourth quartile. The first three quartiles had similar risks of BPH diagnosis.

Results of the sensitivity analysis are presented in Tables 4 and 5. Among the comparison veterans the risk of BPH diagnosis consistently decreased with increasing serum TCDD levels on both continuous and categorical analysis (Table 4). Among the Ranch Hand veterans the risk of BPH diagnosis appeared to increase with increasing TCDD levels when TCDD was used as a continuous variable (Table 5). On categorical analyses with the first quartile of the comparison veterans as the referent category, the increased risk of BPH diagnosis was confined completely to the highest TCDD quartile among the Ranch Hand veterans. The first three quartiles of the Ranch Hand veterans had a decreased risk (not statistically significant) of being diagnosed with BPH compared with the referent category (Table 5, Figure 1).

**Figure 2:**
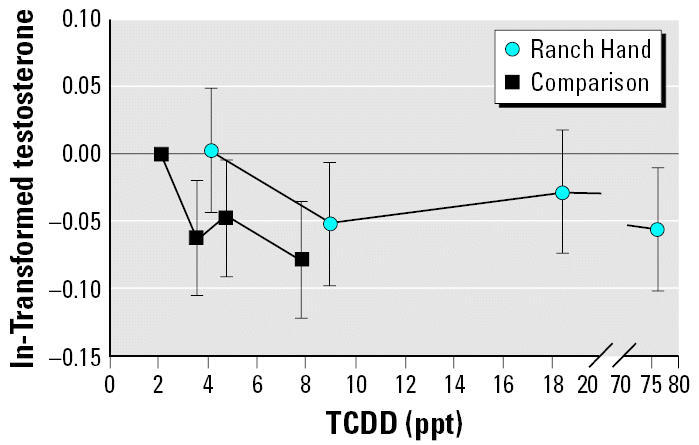
Association of ln-transformed serum testosterone levels (measured in 1987) with quartiles of serum TCDD levels after controlling for age, BMI, and the percentage change in BMI per year among comparison and Ranch Hand veterans in the Air Force Health Study. TCDD quartile I of the comparison veterans is the referent group for the Ranch Hand veterans. The *y*-axis is the linear regression coefficient for the TCDD quartiles; error bars indicate 95% CI.

We also examined the relationship between serum TCDD levels and serum testosterone levels. In multivariate linear regression analysis (Table 6), serum testosterone was negatively associated with serum dioxin levels in both the comparison and the Ranch Hand veterans. Further analyses were performed using the TCDD quartiles, with the first quartile of the comparison veterans serving as the referent category. A consistent decrease in serum testosterone was seen across all TCDD quartiles for both Ranch Hand and comparison veterans (Table 7, Figure 2).

## Discussion

In this prospective cohort study, higher serum TCDD levels in the comparison group are associated with decreased risk of being diagnosed with BPH. Serum TCDD is also associated with lower testosterone levels in both Ranch Hand and comparison veterans.

The TCDD exposure levels in the comparison group are similar to the ‘background’ exposure levels in the general population (4.22 ppt according to the 1987 NHATS) (Orban et al. 1994). These results are consistent with the results of our previous cross-sectional study in which we found a decrease in the odds of having BPH with increasing TCDD body burden at general population exposure levels (Gupta et al. 2006). To our knowledge, the present study is the only prospective study that has examined the association between serum TCDD and BPH.

In the present study we showed an inverse association between serum testosterone and TCDD levels. Other investigators have also reported similar results (Egeland et al. 1994; Johnson et al. 2001). Egeland et al. (1994) studied 231 controls and 248 chemical production workers who were occupationally exposed to TCDD and found an inverse association between TCDD and serum testosterone. Johnson et al. (2001), in their study of 37 workers exposed to TCDD through spraying of herbicides, found a statistically significant inverse relationship between TCDD and testosterone in some of their analyses. The present study has the largest sample size compared to prior studies that have investigated the same hypothesis.

The strengths of our study are that it was prospective in nature and the loss to follow-up was minimized. We included two groups: the comparison veterans and the Ranch Hand veterans. The comparison veterans were exposed to the background exposure levels in the general population, whereas the Ranch Hand group was exposed to the background level plus a varying amount of TCDD through exposure to Agent Orange. This enables us to study the effects of dioxin exposure in two comparable populations with two different mechanisms of exposure. The prospective nature of this study resolves the temporal ambiguity inherent in cross-sectional studies because the serum dioxin levels were measured before the veterans were diagnosed with BPH. More than half of the participants—56% of the comparison veterans and 57% of the Ranch Hand veterans—had experienced the outcome of interest (i.e., had been diagnosed with BPH over the follow-up period). This provides our study with adequate statistical power to evaluate the relationship between serum TCDD levels and BPH. The results of the study were consistent when different exclusion criteria were applied (Tables 4 and 5).

Our study is limited by the fact that serum levels were measured only for TCDD and we did not have data on the levels of other dioxin and dioxin-like congeners. TCDD was the major dioxin in Agent Orange, and other dioxin-like compounds were not considered in the initial study design. In the general population, TCDD accounts for < 5% of the total dioxin toxic equivalents in the body (Schecter and Gasiewicz 2003). The study population was predominantly composed of whites; thus, the results may not be generalized to the entire population. BPH was determined by use of medical records, which may result in some misclassification. However, any misclassification is unlikely to be differential with respect to serum TCDD levels and thus is expected to bias the study results toward the null.

BPH was assessed as a dichotomous outcome in this study. Correlating a continuous outcome measure such as prostate volume with TCDD levels is expected to be a more sensitive measure of the effect of dioxins on prostate growth. Although prostate volume measurement is invasive, it merits consideration for further studies. The data we used for assessing the relationship between testosterone and TCDD is cross-sectional in nature because both TCDD and testosterone were measured in 1987. Thus, the results represent associations and do not prove causation.

Prostatic growth in rats is sensitive to TCDD exposure (Appendix). The mechanisms of the effect of TCDD exposure on rat prostate might help in explaining the observed association between TCDD exposure and BPH (Appendix).

TCDD is also known to decrease testosterone levels in adult male rats. In adult rats exposed to TCDD, testosterone decreased in a dose-dependent fashion, and there was a dose-dependent decrease in volume per testis of Leydig cell cytoplasm, nuclei, or total Leydig cell volume (Johnson et al. 1992). TCDD exposure also decreased the number of Leydig cells, size of individual Leydig cells and the volume per testis of smooth endoplasmic reticulum and mitochondria (Johnson et al. 1994). Moreover, TCDD also inhibits the compensatory rise in the concentration of luteinizing hormone in plasma in response to low testosterone levels in rats (Bookstaff et al. 1990a, 1990b).

The difference in the results for the comparison and the Ranch Hand groups with respect to the association between serum TCDD levels and the risk of being diagnosed with BPH is surprising and not readily explainable. The results for the quartiles I–III of the Ranch Hand veterans were consistent with the results of the comparison group (Figure 1). However, the quartile IV showed an increased risk that was not statistically significant when compared with the referent category, but it was statistically significant if the comparison veterans were not used as the referent group and TCDD was treated as a continuous variable (Table 3). Also, there was a statistically significant trend toward higher risk of BPH with increasing TCDD levels when certain exclusion criteria were applied (Table 5). This increased risk was confined exclusively to the TCDD quartile IV. The reason for this finding is not known. The finding may have occurred due to chance, but a few alternate explanations are also plausible.

First, the results are almost U-shaped, with a decrease in risk followed by an increasing risk on BPH. Other investigators studying endocrine-active chemicals have also noticed such results whereby the initial increase or decrease was followed by a subsequent reversal. For example, Eskenazi et al. (2005) studied the risk of early menopause with exposure to TCDD and found a nonmonotonic dose-related association. They divided the data into quintiles based on serum TCDD levels. The risk ratio for the second, third, and fourth quintiles compared with the first quintile was 1.1, 1.4, and 1.6, respectively (test for trend, *p* = 0.04); but for the fifth quintile the risk ratio was 1.1. Similarly, in another study (Markowski et al. 2001), a curvilinear association between body weight and TCDD dose was seen in both male and female Holtzman rats; the body weight of rats exposed to lower dioxin doses (20 ng/kg and 60 ng/kg) was higher than in controls and rats exposed to a higher TCDD dose (180 ng/kg). Hormones and endocrine-disrupting chemicals are thought to have a U- or inverted U-shaped response because lower concentrations of a hormone can stimulate a tissue, whereas higher concentrations can have the opposite effect (vom Saal et al. 1997). Mice exposed to lower concentrations of estradiol or diethylstilbestrol had higher prostate weights compared with controls and mice exposed to higher concentrations of estradiol and diethylstilbestrol (vom Saal et al. 1997). Similarly, lower concentrations of bisphenol A (an estrogenic compound) produced greater increases in body weight and uterine weight than higher doses (Rubin et al. 2001). Other studies have also shown similar trends (vom Saal et al. 1995, 1998). Thus, it is possible that the effect of TCDD exposure on the human prostate follows a U-shape, whereby the initial decrease in BPH with lower doses is followed by increased occurrence of BPH at higher doses.

Second, the mechanism of exposure to TCDD differs between the comparison and Ranch Hand veterans. The comparison group was exposed to continuous background levels of dioxins, whereas the Ranch Hand group was exposed to a “bolus” of dioxins (specifically TCDD) while involved in the spraying of Agent Orange, in addition to exposure to background levels of dioxins. A possible explanation of the observed difference is that the reproductive effects of dioxins may be most pronounced when exposure occurs earlier in life. Thus, the background exposure levels at an early age may have a greater influence than a bolus TCDD exposure later. We consider the serum TCDD levels in the comparison group representative of the exposure levels experienced at a much younger age; however, the TCDD levels in the Ranch Hand group are sums of background exposure and bolus exposure from TCDD-contaminated Agent Orange. This bolus exposure may have masked the effects of the earlier background exposure and would make assessing the effects of TCDD exposure difficult. This difference in the mechanism of TCDD exposure may explain why the steady decrease in risk of BPH observed in the comparison group is not seen in the Ranch Hand veterans. Evidence from prior studies shows that age at TCDD exposure is an important determinant of the effects. The median effective dose (ED_50_) of TCDD that produces decreases in testosterone and dihydrotestosterone levels in adult rats is 15 μg/kg TCDD (Moore et al. 1985), whereas the ED_50_ for *in utero* and lactational TCDD exposure of 0.16 μg/kg TCDD can produce a spectrum of adverse effects such as decreased weight of ventral prostate and seminal vesicles and decreased epididymal sperm numbers (Mably et al. 1992). Hardell et al. (2003) reported that mothers of men with testicular cancer had higher PCB levels than controls. The men themselves did not have high PCB levels. This suggests that TCDD exposure during development is more predictive of future outcomes. Further studies examining age in relation to TCDD exposure and future outcomes are needed.

## Conclusions

TCDD exposure is associated with effects on the human prostate. The risk of BPH decreases with increasing TCDD exposure, but may increase at higher doses. Also, TCDD exposure is negatively associated with serum testosterone levels.

## Correction

In the original manuscript published online, “BMI at the start of the Southeast Asia tour” was incorrect. It has been corrected here (in the text and in Tables 1, 4, and 5). “BMI_tour_” is now defined as the BMI at the end of the Southeast Asia tour. Veterans were excluded from the study if the BMI was not available for the end of the Southeast Asia tour.

## Figures and Tables

**Table 1:** Descriptive characteristics of comparison (*n* = 1,259) and Ranch Hand (*n* = 964) veterans in the Air Force Health Study.

Characteristic	Comparison	Ranch Hand
Age (years)^a^	48.8 ± 0.2	48.9 ± 0.2
Age at end of tour (years)	30.0 ± 0.2	29.8 ± 0.2
TCDD (ppt)	4.57 ± 0.08	26.93 ± 1.47
ln(TCDD)	1.38 ± 0.02	2.63 ± 0.04
ln(testosterone)^a^	6.24 ± 0.01	6.26 ± 0.01
BMI^a^	27.7 ± 0.11	27.5 ± 0.13
Percentage change in BMI per year	0.60 ± 0.02	0.60 ± 0.02
Race [*n* (%)]		
Black	77 (6.1)	57 (5.9)
White	1,182 (93.9)	907 (94.1)

Values shown are mean ± SE except where noted.

a Values at the 1987 examination.

**Table 2:** Distribution of the study population into quartiles based on serum TCDD levels.

	TCDD			
Group/TCDD quartile	Range (ppt)	Mean TCDD (ppt)	N_BPH_/N_tot_	Testosterone [mean ± SD (ng/mL)]
Comparison				
I	0.42–2.97	2.14	167/319	606 ± 191
II	2.98–4.08	3.54	177/309	534 ± 153
III	4.09–5.53	4.74	172/315	517 ± 152
IV	5.54–54.8	7.87	189/316	491 ± 142
Total			705/1,259	537 ± 166
Ranch hand				
I	0.57–6.50	4.14	137/240	583 ± 157
II	6.51–11.97	8.95	158/247	527 ± 167
III	11.98–26.69	18.40	131/235	542 ± 171
IV	26.70–617.75	76.16	125/242	530 ± 154
Total			551/964	545 ± 164

Abbreviations: N_BPH_, number of men with BPH in each quartile; N_tot_, total number of men in the quartile.

**Table 3:** RR (95% CI) of developing BPH with increasing serum TCDD levels among comparison and Ranch Hand veterans in the Air Force Health Study.

	Comparison		Ranch Hand	
Method	Univariate RR (95% CI)	Multivariate RR (95% CI)	Univariate RR (95% CI)	Multivariate RR (95% CI)
By continuous variable				
ln(TCDD)	0.78 (0.68–0.89)	0.84 (0.73–0.98)	1.07 (0.98–1.16)	1.12 (1.03–1.22)
ln(testosterone)^a^	1.55 (1.21–2.00)	1.41 (1.07–1.84)	1.59 (1.19–2.11)	1.47 (1.08–2.00)
BMI	0.98 (0.96–1.00)	0.98 (0.95–1.00)	0.98 (0.96–1.01)	0.95 (0.93–0.98)
Percentage change in BMI per year	1.07 (0.95–1.20)	1.22 (1.05–1.41)	1.10 (0.96–1.26)	1.33 (1.12–1.57)
By quartile				
TCDD quartiles				
I	1*	1**	0.84 (0.67–1.06)#	0.85 (0.68–1.07)##
II	0.91 (0.74–1.13)	0.96 (0.78–1.20)	0.83 (0.67–1.04)	0.91 (0.73–1.13)
III	0.75 (0.61–0.93)	0.81 (0.65–1.01)	0.79 (0.63–0.99)	0.91 (0.72–1.15)
IV	0.67 (0.54–0.83)	0.76 (0.61–0.95)	1.02 (0.81–1.28)	1.13 (0.89–1.44)
ln(testosterone)^a^	1.55 (1.21–2.00)	1.40 (1.07–1.84)	1.59 (1.12–2.11)	1.29 (0.99–1.68)
BMI	0.98 (0.96–1.00)	0.98 (0.95–1.00)	0.98 (0.96–1.00)	0.96 (0.93–0.98)
Percentage change in BMI per year	1.07 (0.95–1.20)	1.20 (1.03–1.39)	1.10 (0.96–1.26)	1.33 (1.14–1.54)

Analyses were performed by treating ln(TCDD) as a continuous variable and also by dividing TCDD into quartiles. TCDD quartile I of the comparison veterans is the referent group for other TCDD quartiles in both comparison and Ranch Hand veterans.

a Values at the 1987 examination.

* *p* < 0.001,

** *p* = 0.049,

# *p* = 0.12, and

## *p* = 0.18 for trend across TCDD categories.

**Table 4:** RR (95% CI) of developing BPH with increasing serum TCDD categories among comparison veterans in the Air Force Health Study.

				RR^a^ (95% CI) by quartile				
Excluded	BPH cases (*n*)/total subjects (*n*)	RR^a^ (95% CI)	*p*-Value	I	II	III	IV	*p*-Value for trend
Men with history of prostate cancer	650/1,186	0.84 (0.73–0.98)	0.02	1	0.94 (0.75–1.18)	0.78 (0.62–0.98)	0.76 (0.61–0.96)	0.05
Men with history of prostate cancer, inflammatory prostatic diseases, or other prostatic diseases	556/1,047	0.84 (0.71–0.99)	0.03	1	0.96 (0.76–1.22)	0.80 (0.62–1.02)	0.79 (0.61–1.02)	0.14
Men diagnosed with BPH prior to 1988	544/1,097	0.84 (0.71–0.99)	0.03	1	0.95 (0.74–1.21)	0.79 (0.61–1.01)	0.74 (0.57–0.96)	0.06
Men diagnosed with BPH prior to 1991	526/1,037	0.85 (0.72–1.01)	0.06	1	0.98 (0.76–1.25)	0.80 (0.62–1.03)	0.77 (0.59–1.00)	0.09
Men diagnosed with BPH prior to 1994	381/879	0.77 (0.64–0.94)	0.01	1	0.97 (0.73–1.28)	0.70 (0.52–0.94)	0.66 (0.49–0.90)	0.01
Men diagnosed with BPH prior to 1994 and men with history of prostate cancer, inflammatory prostatic diseases, or other prostatic diseases	330/767	0.75 (0.61–0.93)	0.01	1	0.98 (0.73–1.33)	0.69 (0.50–0.95)	0.68 (0.49–0.94)	0.02

a Adjusted for testosterone levels in 1987, BMI in 1987, and the percentage change in BMI per year since the end of the Southeast Asia tour.

**Table 5:** RR (95% CI) of developing BPH with increasing serum TCDD categories among Ranch Hand veterans in the Air Force Health Study.

				RR^a^,^b^ (95% CI) by quartile				
Excluded	BPH cases (*n*)/total subjects (*n*)	RR^a^ (95% CI)	*p*-Value	I	II	III	IV	*p*-Value for trend
Men with history of prostate cancer	512/907	1.11 (1.02–1.21)	0.02	0.86 (0.68–1.09)	0.95 (0.76–1.19)	0.89 (0.70–1.14)	1.14 (0.89–1.45)	0.23
Men with history of prostate cancer, inflammatory prostatic diseases, or other prostatic diseases	448/819	1.11 (1.02–1.21)	0.02	0.92 (0.72–1.18)	0.98 (0.76–1.25)	0.93 (0.71–1.22)	1.22 (0.94–1.59)	0.23
Men diagnosed with BPH prior to 1988	403/816	1.18 (1.07–1.30)	0.001	0.81 (0.62–1.05)	0.79 (0.61–1.02)	0.82 (0.62–1.08)	1.20 (0.92–1.57)	0.01
Men diagnosed with BPH prior to 1991	394/785	1.19 (1.07–1.31)	0.001	0.82 (0.63–1.07)	0.80 (0.62–1.05)	0.85 (0.64–1.12)	1.24 (0.94–1.63)	0.01
Men diagnosed with BPH prior to 1994	272/657	1.19 (1.05–1.33)	0.005	0.74 (0.54–1.01)	0.78 (0.58–1.06)	0.73 (0.52–1.03)	1.16 (0.84–1.59)	0.02
Men diagnosed with BPH prior to 1994 and men with history of prostate cancer, inflammatory prostatic diseases, or other prostatic diseases	242/585	1.14 (1.01–1.29)	0.04	0.81 (0.58–1.12)	0.90 (0.65–1.25)	0.77 (0.53–1.12)	1.17 (0.84–1.64)	0.16

a Adjusted for testosterone levels in 1987, BMI in 1987, and the percentage change in BMI per year since the end of the Southeast Asia tour.

b TCDD quartile 1 of the comparison veterans was the referent group.

**Table 6:** Association of serum testosterone (ln-transformed) with serum TCDD levels after controlling for age, BMI, and the percentage change in BMI per year among comparison (*n* = 1,259) and Ranch Hand (*n* = 964) veterans in the Air Force Health Study.

	Comparison [coefficient (95% CI)]	Ranch Hand [coefficient (95% CI)]
ln(TCDD)	−0.05 (−0.08 to −0.03)	−0.02 (−0.04 to −0.002)
Age^a^	−0.01 (−0.01 to −0.01)	−0.01 (−0.01 to −0.01)
BMI^a^	−0.02 (−0.03 to −0.02)	−0.02 (−0.03 to −0.02)
Percentage change in BMI per year	−0.040 (−0.069 to −0.010)	−0.024 (−0.058 to 0.011)

a Values at the 1987 examination.

**Table 7:** Association of serum testosterone (ln-transformed) levels (measure in 1987) with quartiles of serum TCDD levels after controlling for age, BMI, and the percentage change in BMI per year among comparison and Ranch Hand veterans in the Air Force Health Study.

TCDD quartiles	Coefficient (95% CI)	*p-*Value
Comparison		
I	0^a^ (—)	—
II	−0.063 (−0.105 to −0.012)	0.004
III	−0.048 (−0.091 to −0.005)	0.03
IV	−0.079 (−0.123 to −0.036)	< 0.001
Ranch hand		
I	0.002 (−0.044 to 0.047)	0.94
II	−0.052 (−0.098 to −0.007)	0.03
III	−0.029 (−0.075 to 0.017)	0.22
IV	−0.056 (−0.102 to −0.10)	0.02

a TCDD quartile I of the comparison veterans is the referent group for other TCDD quartiles for both comparison and Ranch Hand veterans.

## Appendix

**Table:**

**Biology of BPH**	**Effect of TCDD on Rat Prostate**
BPH may be caused by embryonic reawakening of prostatic stromal cell inductive potential (McNeal 1978).	TCDD inhibits prostate growth on intrauterine exposure (Roman et al. 1995; Theobald and Peterson 1997).
The number of epithelial and stromal cells increase in BPH.	TCDD inhibits and delays differentiation of prostatic luminal epithelial cells and pericordial smooth muscle cells (Roman et al. 1998; Theobald et al. 2000).
BPH nodules originate through ductal budding (McNeal 1978).	TCDD decreases the number of buds, ductal tips, and main ducts and inhibits branching morphogenesis of all prostate lobes (Roman et al. 1998; Timms et al. 2002) without impairing the AR signaling pathway (Ko et al. 2004).
Human prostate expresses androgen receptors (ARs) throughout life (Barrack et al. 1983).	TCDD decreases AR expression in the ventral prostate (Ohsako et al. 2001; Theobald et al. 2000).
Androgens are required for normal cell proliferation and differentiation.	TCDD decreases androgen-responsive mRNA expression in the ventral prostate (Roman and Peterson 1998).
Development of BPH requires androgens during prostate development, puberty, and aging (McConnell 1995).	
Nuclear AR levels may be higher in BPH tissue than in normal tissue (Barrack et al. 1983).	TCDD decreases formation of androgen-responsive prostatic epithelial cells (Theobald et al. 2000).
The prostate completely fails to develop in testicular feminization syndrome in which ARs are defective or completely absent.	These effects are not explained by decreased testicular androgen production or by decreased conversion to dihydrotestosterone (Roman et al. 1995; Timms et al. 2002).
